# Maxillary Sinus Floor Augmentation to Enable One-Stage Implant Placement by Using Bovine Bone Substitute and Platelet-Rich Fibrin

**DOI:** 10.1155/2018/6562958

**Published:** 2018-08-13

**Authors:** Horia Mihail Barbu, Claudia Florina Andreescu, Monica Raluca Comaneanu, Daniel Referendaru, Eitan Mijiritsky

**Affiliations:** ^1^Department of Specialties in Dental Medicine, Faculty of Dental Medicine, Titu Maiorescu University, Bucharest 031593, Romania; ^2^Oral and Maxillofacial Surgery, Department of Otolaryngology, Tel-Aviv Sourasky Medical Center, Sackler Faculty of Medicine, Tel-Aviv University, Tel-Aviv 64239, Israel

## Abstract

Nowadays it is possible to perform an optimal implant placement and to achieve a good long-term prognosis for an implant-borne prosthesis in the grafted posterior maxilla. This study evaluates the efficiency of one-stage piezosurgery by using as graft material a combination of particulate bovine bone substitutes with platelet-rich fibrin to achieve sinus lift. We included in this study 14 cases of one-stage sinus lift surgeries during which we placed 30 standard implants. The mean vertical bone height gain was 10.12 mm six months after surgery, and the mean postoperative follow-up time was 43.79 months. There were no major complications during or after surgery, and all implants are in use. Therefore, it can be concluded that one-stage sinus piezosurgery using particulate bovine bone substitutes and platelet-rich fibrin can be applied as a predictable and effective technique in the treatment of the posterior edentulous maxilla ensuring 4-5 mm vertical bone height.

## 1. Introduction

Maxillary sinus floor augmentation (also known as sinus lift, sinus graft, sinus augmentation, or sinus procedure) is a surgical procedure, which increases the amount of bone in the posterior maxilla by the elevation of the sinus (Schneiderian) membrane from the underlying sinus wall and by placing a bone graft under it. The aim of sinus augmentation is to obtain bone to support a dental implant. Implants can be applied at the same time as sinus surgery (simultaneous placement) or after a healing period (delayed placement).

Since 1974 when the first surgery of sinus lift was performed, the science of biomaterials has improved by enhancing the possibilities of graft augmentation and allowing clinicians to perform implant-borne dental restorations in complex situations. As a result, it is possible to perform an optimal implant placement and to achieve a good long-term prognosis for an implant-borne prosthesis in the posterior grafted maxilla. Currently, maxillary sinus augmentation is a well-documented surgery with long-term clinical success/survival of the implants similar to those placed in the pristine bone [1–3].

However, there is a debate about the best biomaterial or combination of biomaterials regarding sinus surgery. Studies reported that implants placed in the sinuses augmented with particulate grafts presented a higher survival rate than those augmented with block grafts [4]. Bovine bone mineral acts as a slowly resorbing space maintainer [5] and can diminish sinus pneumatisation after augmentation. Platelet-rich fibrin (PRF) [6] is a fibrin concentrate obtained from the patient's blood, with integrated growing factors and cytokines, which provides a favourable environment for cell migration and rapid vascularization [7]. Studies showed that PRF promotes bone healing and could increase the success rate of bone grafting [8, 9].

The association of particulate bovine bone graft with PRF could allow faster healing and earlier rehabilitation. The purpose of this study was to evaluate one-stage piezosurgery using as graft material a combination of particulate bovine bone substitutes with PRF to attain sinus lift.

## 2. Materials and Methods

This study comprises the cases of 14 patients who required sinus augmentation. The study was conducted in accordance with the standards of the Declaration of Helsinki (1983) and was approved by the Ethical Board of Titu Maiorescu University. The patients were informed about the aim and design of the study and signed a written consent form before surgery. All patients were candidates for maxillary sinus floor augmentation and simultaneous implant placement during October 2013 and June 2014.

Exclusion criteria were as follows: diabetes, hemocoagulation disorders, immunological deficiency, previous radiation therapy of the head-neck area, or patients undergoing treatment with bisphosphonates.

Inclusion criteria were as follows: posterior edentulous subjects with 4-5 mm of crestal bone height, pathology-free sinus, and being without active periodontal diseases. In all cases, the alveolar bone ridge was wide enough for simultaneous implant placement.

Cone beam computed tomography (CBCT) was performed to measure the vertical and horizontal bone height existent between the alveolar crest and the sinus floor and to evaluate the health and anatomy of maxillary sinus [10–12].

The used surgical procedure was lateral window technique (lateral or direct sinus lift) with simultaneous implant(s) insertion. Premedication with antibiotics (Amoxicillin or Clindamycin) was started one day prior to surgery for seven days.

Surgery was performed under local anaesthesia (Articaine 1:100,000 Epinephrine) by applying piezosurgery (Piezosurgery® touch Mectron) to minimize trauma and intraoperative complications [13].

**Figure 1:**
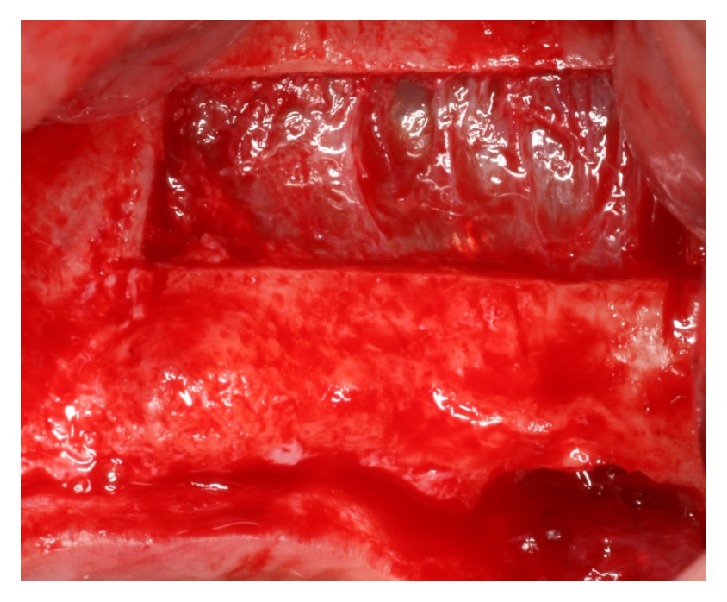
Lateral osteotomy using piezosurgery and the elevation of the sinus membrane.

The modified Caldwell-Luc approach was used to access the maxillary sinus through the lateral wall. A mucosal midcrestal incision was performed with anterior and posterior releasing vestibular incisions certain distance from the proposed osteotomy site. A full-thickness flap was reflected to expose the lateral maxillary wall. An oval or round bony window was created with the piezoelectric instrument so that the Schneiderian membrane became visible (Figure 1). The sinus membrane was elevated carefully with a sinus curette.

**Figure 2:**
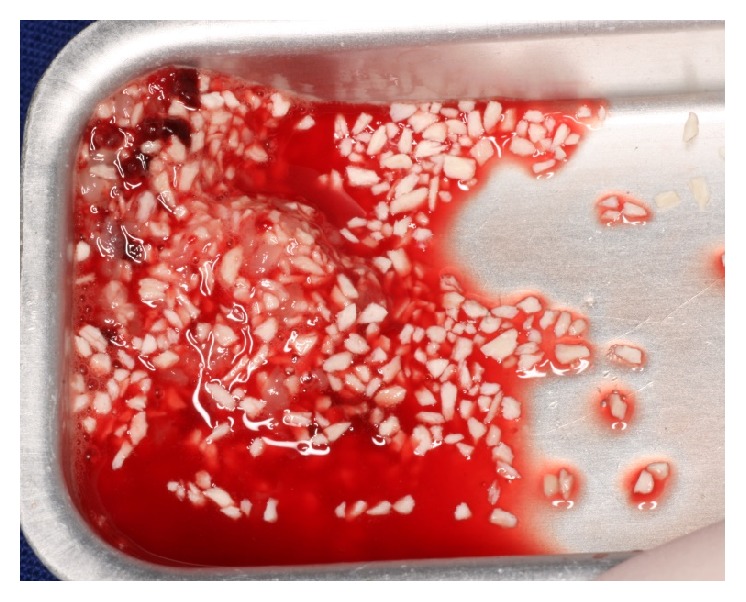
Mixture of xenograft particles and shredded PRF membrane.

**Figure 3:**
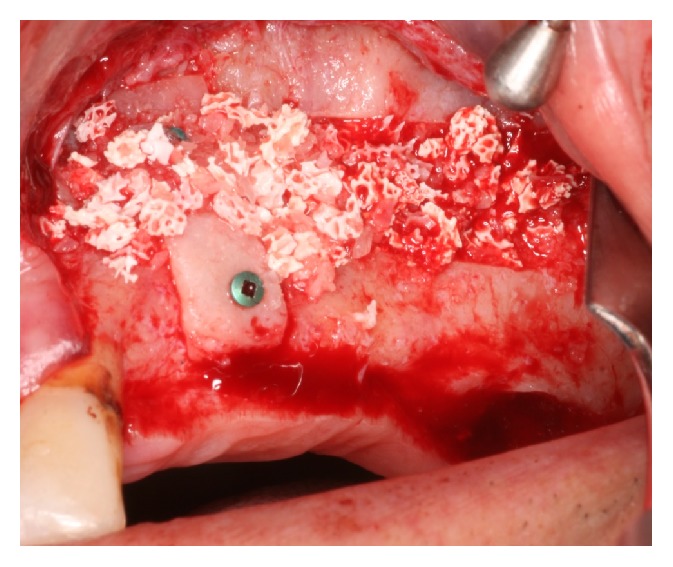
The placement of the mixture in the subsinusal cavity.

Subsequently, the implant sites were prepared, and the cavity between the sinus membrane and the sinus floor was filled in with a mixture of particulate bovine bone graft Bio-Oss® (Geistlich Pharma AG) and autologous PRF (Figures 2 and 3). The implants were inserted when the desired vertical bone height was achieved. The primary stability of implants was verified, and the osteotomy window was covered with the PRF membrane before flap closure.

**Figure 4:**
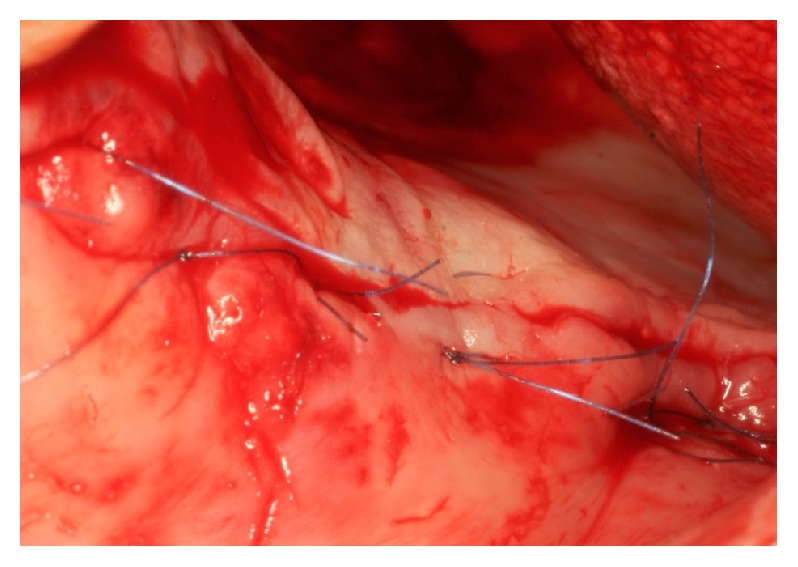
Wound closure.

PRF was obtained according to Choukroun's protocol [14]. The patients' blood samples for PRF preparation were harvested on the same day, before the sinus surgery. The PRF clots were prepared in two different ways: some were transformed in small fragments and mixed with particulate bone substitutes, obtaining an easy-to-use mixture as graft material, and others were transformed in membranes for covering the bone grafting material before wound closure (Figure 4).

**Figure 5:**
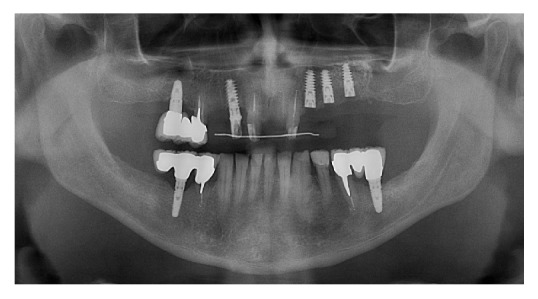
Postoperative X-ray.

**Figure 6:**
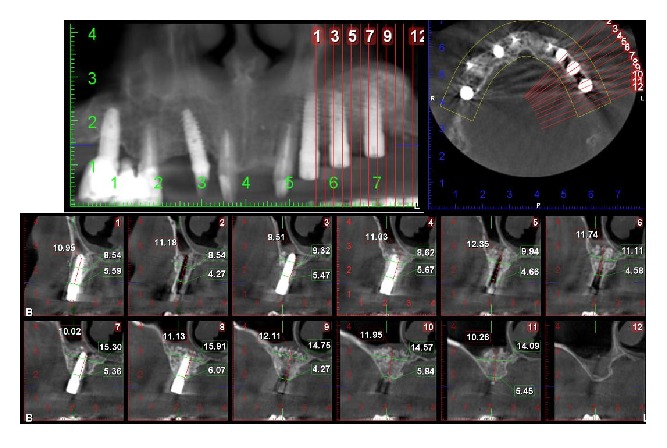
CBCT showing the implants surrounded by dense tissue six months postoperatively.

**Figure 7:**
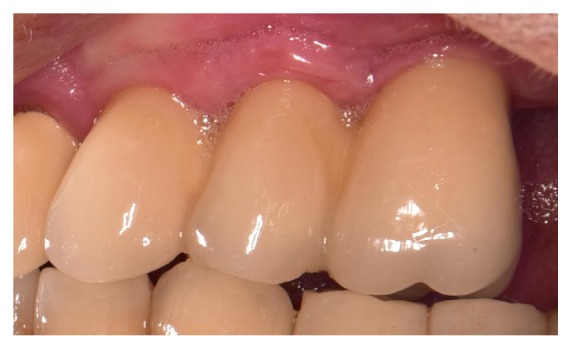
Final restoration.

All patients were followed up after the first week, the first month, three months, and six months postoperatively. The clinical evaluation included the assessment of complications after surgery: pain, oedema, wound dehiscence, graft failure, and implant failure. CBCT or orthopantomography was taken immediately after the intervention (Figure 5). Six months after surgery, a new CBCT was performed to evaluate the bone formation (Figure 6), and prosthetic rehabilitation was started (Figure 7).

The following parameters were assessed: failure of the augmentation procedure, implant failure, major complications of the treated site, vertical bone height, and the duration of treatment starting from surgery to functional loading.

## 3. Results

We performed 14 one-stage sinus lift surgeries and placed 30 standard implants (Table 1). The mean vertical bone height gain was 10.12 mm six months after surgery and the mean postoperative follow-up time was 43.79 months.

Two cases of Schneiderian membrane perforation occurred during surgery (patients nos. 6 and 13). Perforation was closed with PRF clots and membranes, placed directly on the Schneiderian membrane. After the repair of the perforation, sinus augmentation was continued with simultaneous implant placement.

No adverse effects or implant loss was observed in any case during the follow-up period of 6 months or later. Postoperative radiographic assessment revealed the presence of mineralized tissue in all cases without obvious signs of resorption.

## 4. Discussion

Surgeons have three options for grafting the maxillary sinus and implant placement: two-stage lateral sinus augmentation, one-stage lateral sinus augmentation (with simultaneous implant placement), and one-stage crestal approach with simultaneous implant placement, each one with advantages and disadvantages. The choice of surgical technique depends on the quantity and quality of crestal alveolar bone.

According to Kendrick DE 2016 [15], two-stage lateral sinus augmentation is indicated when the crestal bone is less than 3 mm high, one-stage lateral approach when we have 3-4 mm bone height available, and one-stage crestal approach when bone height is above 4-5 mm.

All cases included in the study were based on the lateral technique with simultaneous implantation. All implants were at least 10 mm long and 3.7 mm wide. The functional loading of the implants started six months after surgery, and the prosthetic restorations were cemented crowns.

The lateral approach is considered to be prone to more complications than the crestal one [16] because it is a more invasive technique, but the use of piezoelectric surgery for lateral window preparation and membrane separation led to a dramatic reduction in the occurrence of intraoperative complications [17]. In addition to this, the lateral approach offers better control of the operative site, and it is considered more predictable and useful when extensive implantations are needed [18].

The most common intraoperative complication during sinus surgery is damage to the Schneiderian membrane. Postoperative complications include wound infection, abscess, or dehiscence with drainage, maxillary sinusitis of the surgical site, exposure of the graft, and loss of the graft. Neither of these complications was encountered during the study.

Both biomaterials used in this study are well-documented in the current literature and have multiple applications in oral surgery, but the combination of Bio-Oss® and PRF has been less investigated.

Bio-Oss® is deproteinized bovine bone, frequently used in dental practice to promote bone regeneration because it is biocompatible and osteoconductive and slowly resorbed in humans [19], and it is one of the best-documented biomaterials used in sinus surgery [20]. PRF is an autologous fibrin matrix used to enhance bone regeneration because it can stimulate the proliferation of osteoblasts [21].

Inchingolo et al. 2010 [22] used the association of Bio-Oss® and PRF to treat severe bone maxillary atrophy with vertical bone higher than 5 mm. One-stage sinus surgery was performed in 23 patients with 2-4 mm vertical bone gain and successful prosthetic rehabilitation.

Zhang et al. 2012 [23] assessed the combination of Bio-Oss® and PRF in comparison with Bio-Oss® alone in two-stage sinus lift and reported neither advantages nor disadvantages of the application of PRF in conjunction with deproteinized bovine bone mineral in sinus augmentation after a healing period of six months. On the other hand, it is worth mentioning that adding fibrin gel, like PRF, to particulate bovine bone makes the procedure easier to manage [24].

A combination of Bio-Oss® and PRF in association with second-stage sinus lift and piezosurgery reduced the healing time to 106 days from 150 days [25]. In this study one-stage sinus lift based on piezosurgery and augmentation with Bio-Oss® and PRF was a successful therapy in managing intra- and postoperative complications and prosthetic rehabilitation started six months after surgery, while allogenic–xenogenic sinus graft alone is incomplete six months after the sinus augmentation procedure [26].

PRF alone can be used for sinus floor augmentation as mentioned in several studies [27–31]. When PRF alone is used with simultaneous implant placement vertical bone gain after six months is substantial: 10.1 mm [27], 10.4 mm [28], or 11.8 mm [29]. The histological samples confirmed new bone formation in case of sinus lift with PRF alone in both situations, with and without simultaneous implantations [30], and proved that PRF as a sole graft material during sinus floor augmentation induces natural bone regeneration [31].

According to Nizam et al. 2018 [32], there was no qualitative difference in the histological analyses or the improvement of the amount of regenerated bone when the effect of PRF in combination with deproteinized bovine bone mineral was compared with deproteinized bovine bone mineral alone in maxillary sinus augmentation. Other studies specified the formation of more new vital bone around implants when PRF was added to freeze-dried bone allograft [14] or deproteinized bovine bone mineral [25] in comparison to freeze-dried bone allograft or deproteinized bovine bone mineral alone.

However, PRF as the sole filling material without simultaneous implant placement or particulate bone substitute may not be able to maintain an adequate space under the elevated sinus membrane, because it is resorbable. In these cases, when sinus lift is performed with PRF alone without simultaneous implantation, it is possible that crestal sinus lift is needed during a second surgery for implant insertion.

There is no standardized protocol available for PRF in sinus lift surgery [33], but clot and membrane can be used. Barrier membrane has a positive outcome when considering implant survival after sinus surgery [18, 34]. In this study, the barrier membrane was obtained from PRF concentrate, which had the benefit of being autologous and cheap. The advantage of a PRF membrane is that it stimulates the gingival periosteum and the regeneration of the bone window [35].

Furthermore, the PRF membrane can be used to cover sinus perforation because its self-adherent property eliminates the need for suturing [36–40]. In the present study, the Scheneiderein membrane was perforated in two cases (patients nos. 6 and 13) and subsequently closed with a PRF membrane. The results of the vertical bone augmentation in these two cases were similar to the other cases without sinus membrane perforation.

## 5. Conclusions

One-stage lateral sinus piezosurgery using Bio-Oss® and PRF clot as filling material and PRF membrane as a barrier membrane can be performed as a predictable and effective technique in the treatment of posterior edentulous maxilla with 4-5 mm vertical bone height. The outcome in cases of Schneiderian membrane perforation treated with PRF membrane was similar to the cases without perforation.

## Figures and Tables

**Table 1:** Demonstration of patient data, surgical procedures, and follow-up.

Patient	Age	Gender	Recipient site*∗*	Initial bone height (mm)	Complication during surgery	Major complication after surgery	Control bone height (mm)	Vertical ridge augmentation achieved (mm)	Number of implants	Follow-up (months)*∗∗*
1.	51	M	2.3.; 2.4.; 2.5.	5.00	None	None	11.08	6.08	3	10
2.	46	F	1.4.; 1.5.; 1.6.	4.00	None	None	14.64	10.64	3	20
3.	54	M	1.4.; 1.5.; 1.6.	4.40	None	None	14.10	9.07	3	11
4.	50	F	1.6.	4.49	None	None	15.30	10.81	1	13
5.	42	M	1.6.	4.48	None	None	18.69	14.21	1	13
6.	42	M	2.6.; 2.7.	4.92	Sinusal membrane perforation	None	15.73	10.81	2	27
7.	56	M	1.4.; 1.5.; 1.6.	4.59	None	None	15.93	11.34	3	18
8.	49	M	2.5.; 2.6.	4.89	None	None	14.00	9.11	2	12
9.	42	M	2.6.; 2.7.	4.72	None	None	13.43	8.71	2	12
10.	55	F	1.4.; 1.6.; 1.7.	4.97	None	None	15.39	10.42	3	12
11.	65	M	1.4.; 1.6.	5.00	None	None	15.39	10.39	2	14
12.	46	F	1.4.; 1.5.; 1.6.	4.32	None	None	14.37	10.05	3	13
13.	63	M	1.6.	4.16	Sinusal membrane perforation	None	15.35	11.19	1	14
14.	32	M	2.6.	4.48	None	None	13.36	8.88	1	18
Mean	49.50			4.60	-	-	14.77	10.12	2.14	14.79

*∗*: FDI tooth-numbering system

*∗∗*: after sinus surgery.

## Data Availability

The data used to support the findings of this study are included in the article.
